# Biologically active metabolite(s) from haemolymph of red-headed centipede *Scolopendra subspinipes* possess broad spectrum antibacterial activity

**DOI:** 10.1186/s13568-019-0816-3

**Published:** 2019-06-28

**Authors:** Salwa Mansur Ali, Naveed Ahmed Khan, K. Sagathevan, Ayaz Anwar, Ruqaiyyah Siddiqui

**Affiliations:** Department of Biological Sciences, School of Science and Technology, Sunway University, 47500 Subang Jaya, Selangor Malaysia

**Keywords:** Centipede, Antibacterials, Superbugs

## Abstract

The discovery of novel antimicrobials from animal species under pollution is an area untapped. Chinese red-headed centipede is one of the hardiest arthropod species commonly known for its therapeutic value in traditional Chinese medicine. Here we determined the antibacterial activity of haemolymph and tissue extracts of red-headed centipede, *Scolopendra subspinipes* against a panel of Gram-positive and Gram-negative bacteria. Lysates exhibited potent antibacterial activities against a broad range of bacteria tested. Chemical characterization of biologically active molecules was determined via liquid chromatography mass spectrometric analysis. From crude haemolymph extract, 12 compounds were identified including: (1) l-Homotyrosine, (2) 8-Acetoxy-4-acoren-3-one, (3) *N*-Undecylbenzenesulfonic acid, (4) 2-Dodecylbenzenesulfonic acid, (5) 3*H*-1,2-Dithiole-3-thione, (6) Acetylenedicarboxylate, (7) Albuterol, (8) Tetradecylamine, (9) Curcumenol, (10) 3-Butylidene-7-hydroxyphthalide, (11) Oleoyl Ethanolamide and (12) Docosanedioic acid. Antimicrobial activities of the identified compounds were reported against Gram-positive and Gram-negative bacteria, fungi, viruses and parasites, that possibly explain centipede’s survival in harsh and polluted environments. Further research in characterization, molecular mechanism of action and in vivo testing of active molecules is needed for the development of novel antibacterials.

## Introduction

Given the increasing burden of bacterial infections and multiple-drug resistant bacteria, there is an urgent need for the development of novel antimicrobials (Tacconelli et al. 2018). In the USA alone, at least two million people acquire antibiotic-resistant infections resulting in 23,000 deaths annually (CDC 2018). The rate of emergence of resistant strains is much higher than the rate of introduction of new antibiotics in the market (CDC 2018). The development of antimicrobials from natural products is of prime importance (Mérillon and Rivière 2018; Harvey et al. 2015). Notably, the majority of commercially available natural products are derived from bacteria, fungi and plants. Nearly 70% of antibiotics are derived from soil dwelling bacteria (Smith 2000) such as actinomycin (from *Streptomyces antibioticus*), erythromycin (from *Streptomyces erythraeus*), aminoglycosides (from *Streptomyces* and *Micromonospora*) etc. Likewise, the first antibiotic penicillin was isolated from fungus *Penicillium notatum* (Fleming 1929), cephalosporins from *Acremonium* species (Newton and Abraham 1955) and ascochital, pestalone, indanonaftol A are antibiotics from various fungal species (Bugni and Ireland 2004; Cueto et al. 2001). Similarly, plant and plant products containing sesquiterpenes, triterpenes, flavonoids, procyanidins are shown to possess broad spectrum antibacterial activity against Gram-positive and Gram-negative bacteria (Ahmad et al. 1994). Of note, Kingdom Animalia represents largest diversity with more than 8 million species (Census of Marine Life). Classes such as fishes, amphibians, reptiles, birds and mammals comprises a huge diversity of terrestrial, marine and aquatic fauna (Science daily 2011). Unlike plants, their exposure to polluted environments and disease causing agents is greater. Therefore, it is thought that their ability to defend against pathogenic microorganisms is relevant to humans and must be explored. For example, cockroaches thrive in polluted environments suggesting their innate ability to produce anti-infective agents (Lee et al. 2011). Also, invertebrates particularly insects are used to treat various illnesses and are common in traditional medicines (Costa-Neto 2005). Insects such as hairy arachnids, Chinese black mountain ant, honey bee and bee products, scorpions, grass hoppers, silk worms, termites etc. are believed to possess various health benefits and are used in the treatment of wound healing, pain, cough, inflammation, fever, gastrointestinal related disorders, reproductive illnesses, pneumonia, hemorrhage, diarrhea etc. (Feng et al. 2009; Srivastava et al. 2009). However, the scientific basis of their medicinal properties remains incompletely understood. Previously, we showed the presence of potent antibacterial molecules in cockroaches against methicillin resistant *Staphylococcus aureus* (MRSA) and neuropathogenic *Escherichia coli* K1 (Lee et al. 2011; Ali et al. 2016). Several molecules were identified containing isoquinoline group, chromene derivatives, thiazine groups, imidazoles, pyrrole-containing analogs, sulfonamides, furanones, and flavanones with known antibacterial properties (Ali et al. 2016). Among other species, forest centipede, *Scolopendra subspinipes*, (also named as Vietnamese or Chinese Red-headed centipede) is commonly used in folk medicine, for its various health benefits in the treatment of wounds, pain, inflammation, sores and tumors (Lee et al. 2017; Bajpai et al. 2017; Ding et al. 2016; Choi et al. 2008). Mainly, distributed in East Asian countries, they are large with the maximum length of 20 cm and feeds primarily on insects, arachnids and small vertebrate animals, and encounter pathogens in their natural habitat (Bush et al. 2001). They must have developed mechanisms to counter infections. Hence, we aim to determine antibacterial activity of *S. subspinipes* against a panel of Gram-positive and Gram-negative bacteria and to identify biological molecule(s) using liquid chromatography mass spectrometry.

## Materials and methods

### Bacterial cultures

Eight clinical isolates were tested in this study, among which MRSA (Malaysian Type Culture Collection MTCC 381123), *Bacillus cereus* (MTCC 131621) and *Streptococcus pyogenes* (ATCC 49399) were Gram-positive; while, *Escherichia coli* K1 (MTCC 710859), *Pseudomonas aeruginosa* (American Type Culture Collection ATCC 10145), *Klebsiella pneumonia* (ATCC 13883), *Salmonella enterica* (ATCC 14028) and *Serratia marcescens* (ATCC 13880) were Gram-negative. All the strains were resistant to two or more antibiotics (Table 1). A 24 h old bacterial broth culture was used for experiments as previously described (Khan et al. 2008).

**Table 1:** Antibiotic susceptibility profile of bacteria used in this study

Bacteria/ID no	Antibiotic susceptibility profile									
	amx 25 µg	amc 20/10 µg	cip 10 µg	cst 10 µg	enr 5 µg	gen 10 µg	lcn 15 µg	nxn 10 µg	tcn 30 µg	sxt 1.25 + 23.75 µg
Methicillin-resistant *S. aureus* MTCC 381123	R	R	R	R	R	S	S	R	S	R
*E. coli* K1 MTCC 710859	R	R	S	S	S	S	R	R	S	S
*S. pyogenes* ATCC 49399	R	R	S	R	S	S	R	S	S	I
*B. cereus* MTCC 131621	R	R	S	R	R	S	S	S	S	S
*P. aeruginosa* ATCC 10145	R	R	S	R	R	S	R	S	R	R
*K. pneumonia* ATCC 13883	R	S	S	S	R	S	R	S	R	S
*S. enterica* ATCC 14028	S	S	S	S	S	R	R	S	I	S
*S. marcescens* ATCC 13880	R	R	S	R	S	S	R	S	S	S

### Organ lysates of centipede

**Fig. 1:**
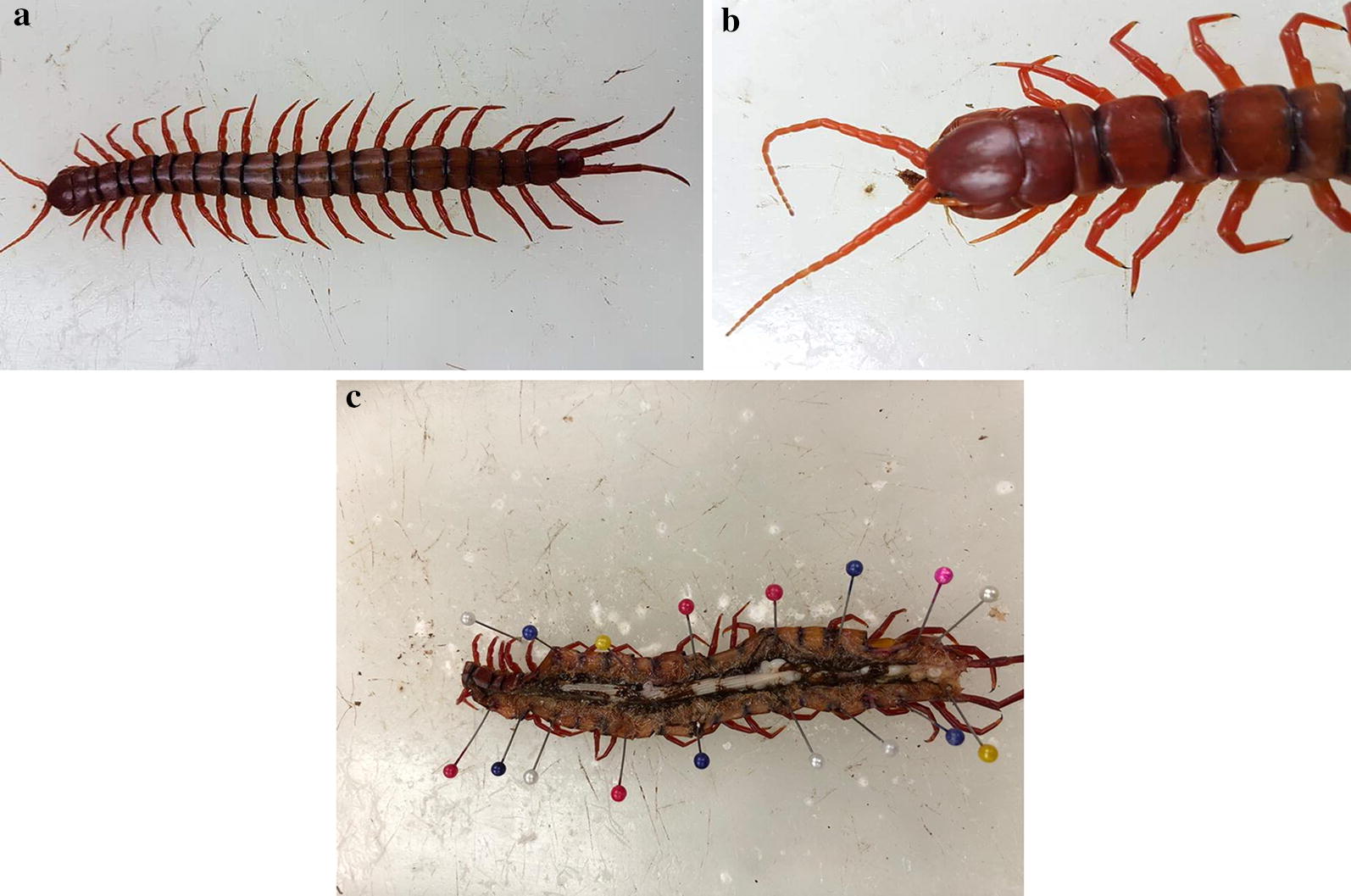
**a** Dorsal view of *S. subspinipes* with intact body segments. **b** Closer view of upper body of centipede. **c** Internal organs of the centipede along the body cavity

Wild forest centipedes (*S. subspinipes*) with approximate length of 18 cm were collected from forest plantation from their natural habitat and kept in a glass cage individually overnight at 30 °C with soil organic matter. 70% ethanol was used to disinfect dissection tools. Centipedes were kept at 4 °C for 15 min. The insect was immobilized by the dissection pins on the anterior and posterior end of the body in a wax tray. The head and legs were removed, and the haemolymph was collected aseptically in ethylenediamine tetraacetic acid (EDTA) containing vacutainer by inserting the sterile pipette tip at the lateral opening of the removed limb (Fig. 1). Digestive system was exposed by the vertical incision made along the midline of the body and the sample was removed aseptically. After collecting the haemolymph and gut, muscle tissue was exposed, a sample of which was aseptically removed and suspended in small volume of sterile distilled water. Protease inhibitors (serine/cysteine/metallo-proteases) were added and the samples were processed at 4 °C and gut and muscle tissue were subjected to ten cycles of freeze-thawing. Homogenization of the samples were performed aseptically with mortar and pestle, followed by sonication and cold centrifugation at 10,000*g* for 30 min. Next, the lysates were filtered with 0.2 μm pore size sterilized filter to avoid contamination and unwanted residual particles, and the protein concentration was determined by Bio-Rad protein assay kit. Lysates were aliquoted and stored at − 20 °C until further usage.

### Antibacterial assay

Antibacterial assays were carried out to determine bactericidal and bacteriostatic activities of haemolymph and tissue lysates of centipede as reported previously (Khan et al. 2008). A 24 h old fresh bacterial culture was adjusted to the absorbance of 0.22 at 595 nm using a spectrophotometer. Approximately 10^6^ bacterial cells suspended in 10 μL of broth, were incubated with 100 µg/mL concentration of organ lysates or 10% haemolymph at 37 °C for 2 h. After incubation, serial dilution of reaction mixture containing bacterial cells was performed followed by plating on nutrient agar plates (Ali et al. 2016; Khan et al. 2008). Bacteria incubated in PBS/broth alone were used as negative control, however, bacteria incubated with 100 μg/mL of gentamicin were used as positive control. Percentage bactericidal/bacteriostatic activity was determined as bacteria surviving relative to the control: 100 − (cfu recovered/original inoculum × 100).

### Human keratinocyte cell (HaCaT) cultures

Human keratinized skin cells (Hacat) (CLS:300493) were purchased from CLS Cell Lines Service, Germany. Cells were cultured in cell culture media comprising RPMI-1640, 10% heat-inactivated fetal bovine serum, 2 mM glutamine, 100 U penicillin/mL, 100 μg streptomycin/mL, non-essential amino acids, and vitamins as previously described (Ali et al. 2016; Khan and Siddiqui 2009). Cell cytotoxicity assays were carried out in 96-well plates by inoculating 5 × 10^5^ HaCaT cells per well per mL followed by incubation at 37 °C with 5% CO_2_ for 48 h. Next, complete monolayer formation was observed microscopically prior to cytotoxicity assays.

### Bacterial-mediated host cell cytopathogenecity assays

Centipede haemolymph (10%) was incubated with 10^6^ bacterial cells at 37 °C for 2 h followed by co-incubation with approx. 2 × 10^6^ HaCaT cells at 37 °C in a 5% CO_2_ incubator for 20 h. Next day, cell suspensions containing metabolites and lactate dehydrogenase enzyme (if present) were collected, centrifuged and subjected to reaction with substrate and dye (present in cytotoxicity detection kit) for 10 min and cytopathogenicity was determined by measuring absorbance of test and control wells at 495 nm. Bacterial-mediated host cell cytopathogenicity were determined and untreated bacteria incubated with human cells were used as controls (Ali et al. 2016; Khan and Siddiqui 2009). Percent cytotoxicity was determined by = (sample value − control value)/(total LDH release − control value) × 100.

### Liquid chromatography–mass spectrometry (LC–MS): separation and analysis

Centipede haemolymph was tested for further chemical identity. Haemolymph was subjected for LC–MS analysis on Agilent 1290 infinity liquid chromatograph (Agilent Technologies, Wilmington, DE), coupled with an Agilent 6520 Accurate-Mass quadrupole-time of flight (Q-TOF) mass spectrometer with dual electrospray ionization source (ESI). Reverse-phase high performance liquid chromatography was used for separation of compounds, with an agilent Zorbax Eclipse XDB-C18, Narrow-Bore 2.1 × 150 mm, 3.5-micron column at 25 °C, and equilibrated with solvent A (0.1% formic acid in Milli-Q water) and solvent B (0.1% formic acid in Acetonitrile). 0.5 mL/min flow rate with a linear gradient was used as follows: 5% solvent B for 5 min, 100% solvent B for 20 min, and 100% solvent B for 25 min. The total run time was 30 min. The compounds were ionized using dual ESI + Accurate-Mass Q-TOF mass spectrometer. The ion source parameters were as follows: capillary voltage at 4000 V for positive and 3000 V for negative ion polarity. Flow rate of drying gas was 10 L/min with a fragmentor voltage of 125 V and gas temperature of 300 °C. Pressure of nebulizer gas was set at 45 psi with Quadrupole-TOF detector, while 50% MeOH + 50% Milli-Q water was used as blank after processing each sample.

### Identification of compounds through Metlin database

As described, haemolymph was processed for liquid chromatography mass spectrometric analysis, in order to obtain the spectra of chromatograms determining molecular mass of the compounds in crude extract. The mass spectra of the compounds retrieved from HPLC were run against Metlin_AM_PCDL-N-170502.cdb for identification with exact homology through Agilent Mass Hunter software, while keeping in view compensation needed for charges in positive ESI MS as well as electron fragmentations, to ensure searches for the correct parent mass. Novelty determination of the identified compounds was performed on Scifinder software. However, previously reported compounds were subjected to literature search for biological activities.

## Results

### Centipede lysates exhibit potent antibacterial activity against broad range of bacteria

**Fig. 2:**
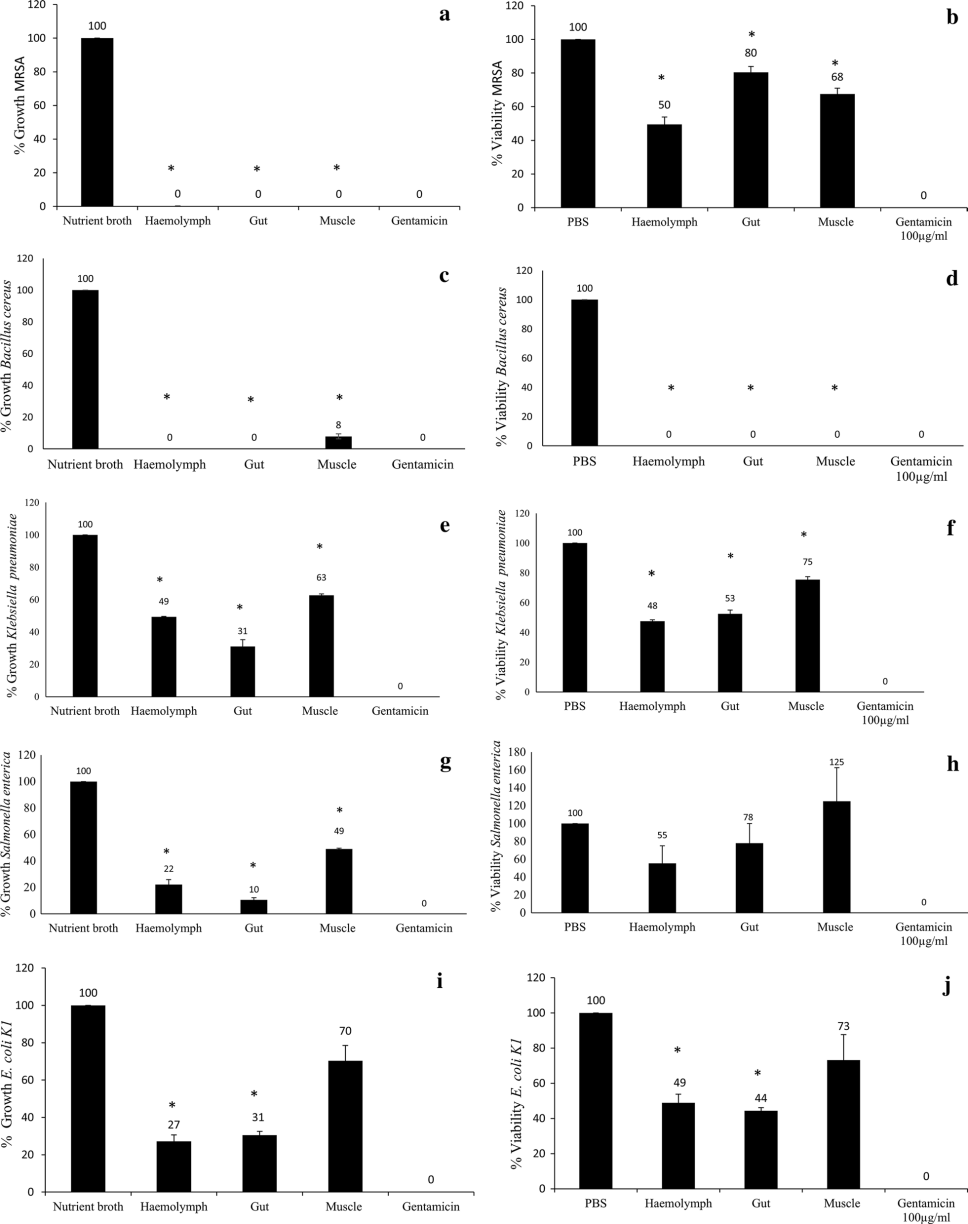

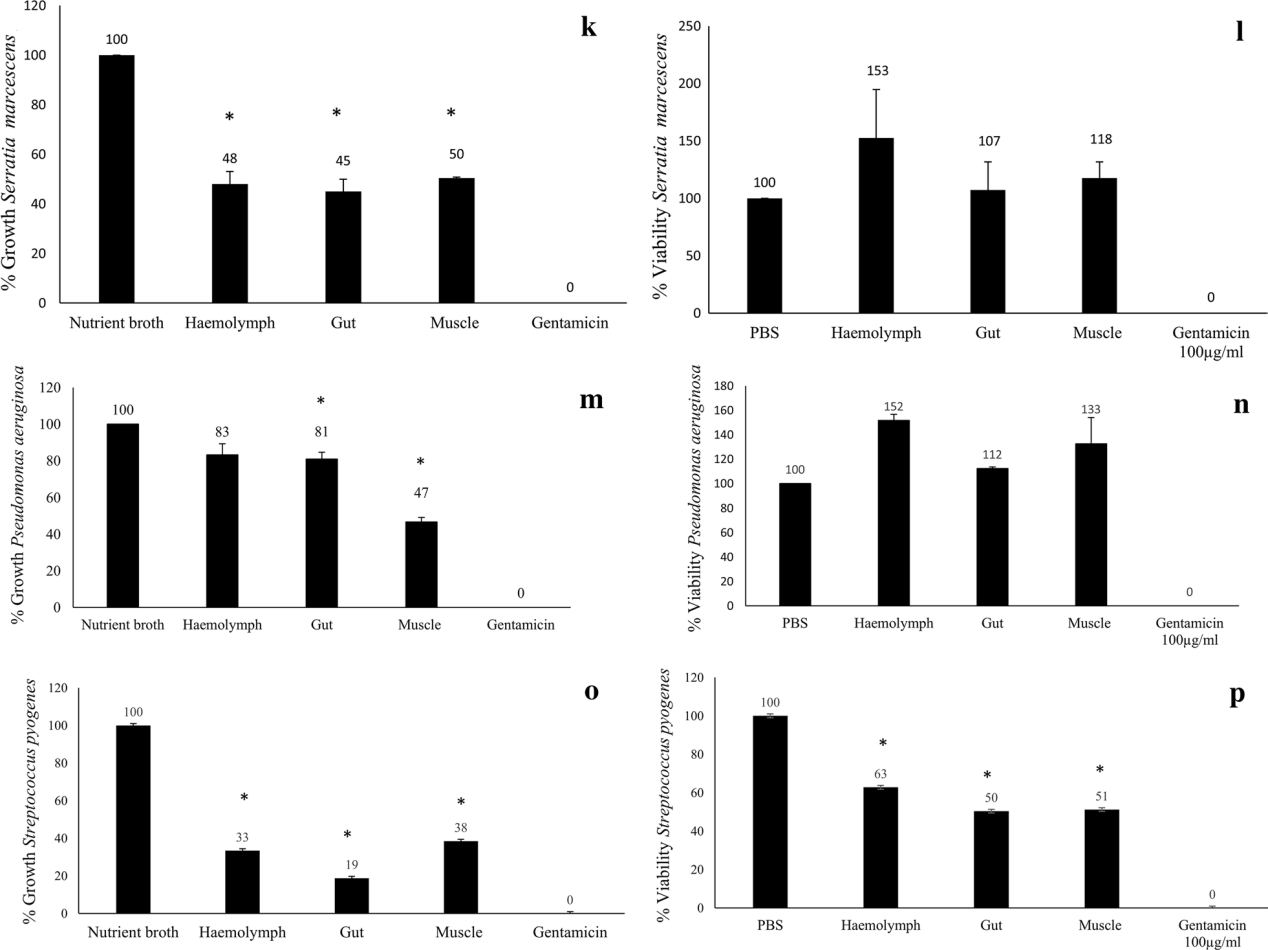
The crude extracts of red centipede’s haemolymph, gut and muscles were prepared and tested in antibacterial bioassays. For negative control, bacteria incubated with nutrient broth/PBS was used and for positive control bacteria incubated with 100 μg/mL of gentamicin was used. Asterisk represents P < 0.05. P values were obtained using two-sample T test and two-tailed distribution. **a** Represents 0% growth indicating potent bacteriostatic activity of 10% haemolymph, 100 μg/mL of muscle and gut extracts of red centipede against MRSA. **b** Represents cidal assay, indicating 50%, 80% and 68% viability of respective extracts against MRSA. **c** Represents more than 90% bacteriostatic activity of all the three extracts against *B. cereus*. **d** Also represents more than 90% bactericidal activity for all three extracts against *B. cereus*. **e** Represents 49%, 31% and 63% growth in bacteriostatic assays respectively against *K. pneumoniae*. **f** Represents 48%, 53% and 75% viability in bactericidal assays respectively against *K. pneumoniae*. **g** Represents 22%, 10% and 49% growth in bacteriostatic assays respectively against *S. enterica*. **h** Represents 55% and 78% viability for haemolymh and gut extracts however, muscle extracts was not active in bactericidal assays against *S. enterica*. **i** Represents 27%, 31% and 70% growth in bacteriostatic assays respectively against *E. coli* K1. **j** Represents 49%, 44% and 73% viability in bactericidal assays respectively against *E. coli* K1. **k** Represents nearly 50% bacteriostatic activity of all three extracts against *S. marcescens*. **l** Represents no bactericidal activity of centipede’s extracts against *S. marcescens*. **m** Represents nearly 83, 81 and 47% growth of centipede’s haemolymph, gut and muscles respectively against *P. aeruginosa*. **n** Represents no bactericidal activity of centipede’s extracts against *P. aeruginosa*. **o** Represents nearly 33, 19 and 38% growth of centipede’s haemolymph, gut and muscles respectively against *S. pyogenes*. **p** Represents 63, 50 and 51% viability of the extracts respectively against *S. pyogenes*. The results are representative of several experiments performed in duplicates and expressed as the mean ± standard error

Centipede’s haemolymph was aspirated and lysates were prepared and tested against Gram-positive and Gram-negative bacteria for determination of antibacterial effects. In particular, haemolymph was remarkably active against bacterial strains tested with more than 90% growth inhibitory activities against MRSA and *B. cereus*, but more than 50% bacteriostatic activity against *E. coli* K1, *K. pneumonia*, *S. enterica*, *S. marcescens* and *S. pyogenes*. Muscle lysates exhibited more than 50% bacteriostatic activity against *S. enterica*, *S. marcescens*, *P. aeruginosa* and *S. pyogenes* (Fig. 2).

### Host cell cytopathogenecity assays

**Fig. 3:**
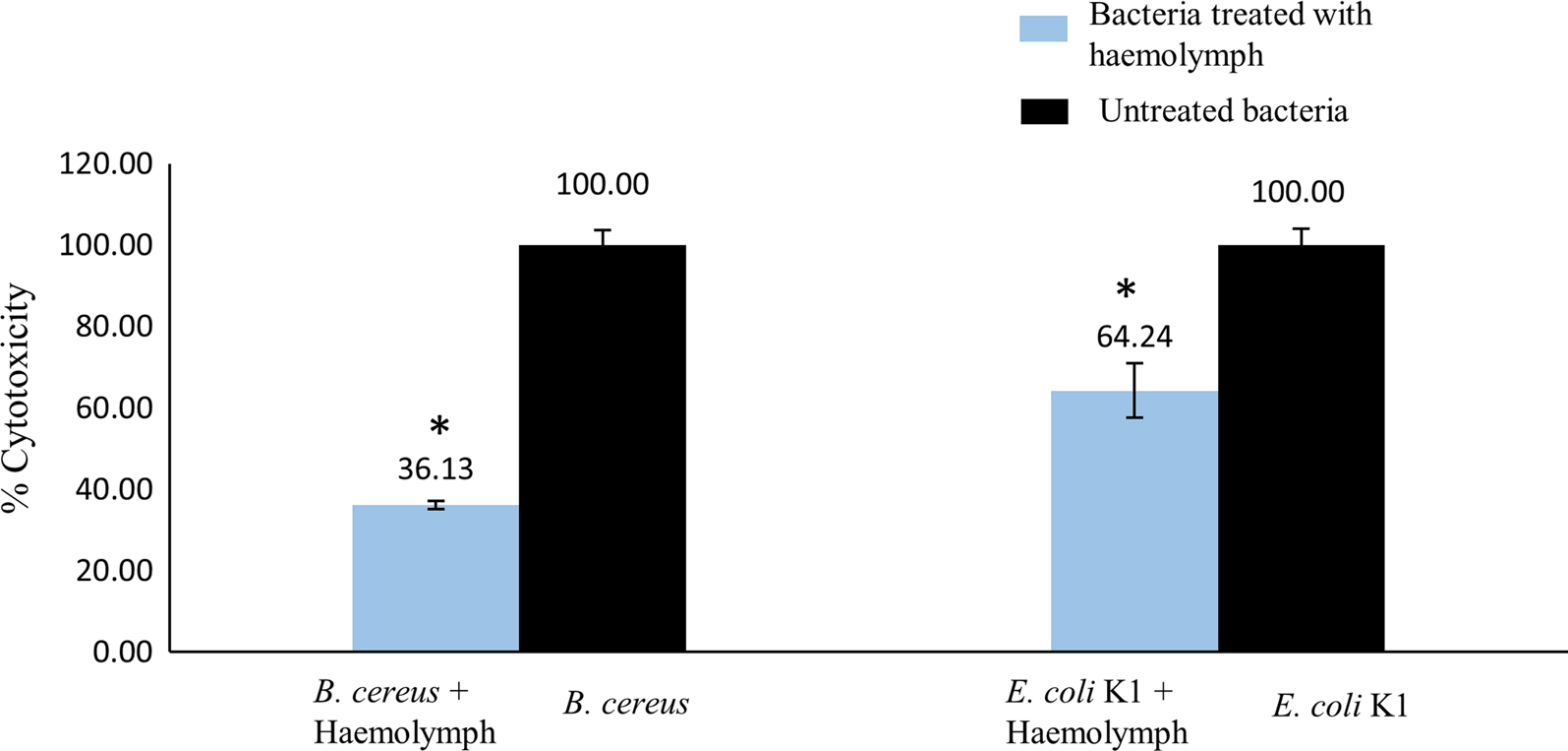
The haemolymph of red centipede was aspirated and tested in cytopathogenicity assay against Human Keratinocyte HaCaT monolayers. 10% haemolymph was incubated with 10^6^ bacterial cells for 2 h at 37 °C, followed by the co-incubation with HaCaT monolayers in 5% CO_2_ incubator at 37 °C for 18–20 h. Untreated bacteria incubated with HaCaT monolayers were used as control. Next day, supernatent containg lactate dehygrogenease enzyme were collected, centrifuged and determined by Rosche cytotocicity detection Kit as per guidelines. Significant reduction was observed in the cytopathogenicity caused by the pre-treated bacteria incubated with haemolymph as compared to untreated bacteria. *B. cereus* showed up to 36% and *E. coli* K1 showed up to 64% cytotoxicity to human cells when pre-treated with haemolymph in contrast to untreated bacteria which showed 100% cytopathogenicity to human cells. The results are representative of several experiments performed in duplicates and expressed as the mean ± standard error

To determine the toxic effects of haemolymph treated bacteria against primary human keratinocytes, cytopathogenicity assays were performed. Treated and untreated bacterial cells were incubated at 37 °C for 2 h, followed by co-incubation with HaCaT monolayers at 37 °C in a 5% CO_2_ incubator for 20 h and lactate dehydrogenase enzyme release (cell lysis marker), was measured using a cytotoxicity detection kit. When treated with 10% haemolymph, *B. cereus* showed host cell death significantly reduced, from 100% to only 36% (P < 0.05). Similarly, *E. coli* K1 treated with haemolymph also showed significant reduction in producing host cell damage (P < 0.05). Notably, haemolymph alone produced approximately 25% host cell damage (data not shown). Overall, the treatment of bacterial cells with centipede’s haemolymph reduced bacterial-mediated host cell damage as compared to untreated bacteria (Fig. 3).

### Identification of biologically active molecule(s) in centipede haemolymph using liquid chromatography–mass spectrometry

**Fig. 4:**
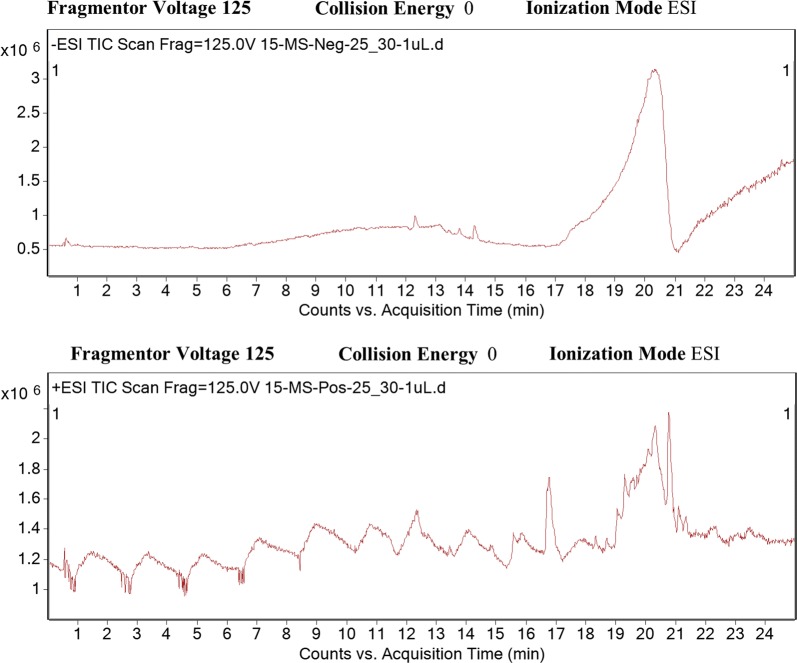
*Scolopendra subspinipes* haemolymph was subjected to LC–MS (Agilent 6520 Accurate-Mass Q-TOF mass spectrometer with dual ESI source) for qualitative analyses. The compounds were separated based on m/z ratio and retention time in the column. The data obtained from the LC–MS for haemolymph contained numerous peaks, out of which 12 compounds have been detected with full identity and molecular structure, however, 36 compounds were detected with the limited information of only molecular mass and retention time in the column

Centipede haemolymph was subjected to LC–MS (Agilent Technologies 6520 Accurate-Mass Q-TOF mass spectrometer with dual ESI source) for qualitative analyses. Figure 4 shows spectra from negative and positive ion polarity. Compounds present in haemolymph were separated in the column on the basis of mass to charge ratio (m/z) and retention time. The data obtained from the LC–MS for haemolymph contained 48 compounds in total, out of which identity of 12 compounds was confirmed. These include, (1) l-Homotyrosine, (2) 8-Acetoxy-4-acoren-3-one, (3) *N*-Undecylbenzenesulfonic acid, (4) 2-Dodecylbenzenesulfonic acid, (5) 3H-1,2-Dithiole-3-thione, (6) Acetylenedicarboxylate, (7) Albuterol, (8) Tetradecylamine, (9) Curcumenol, (10) 3-Butylidene-7-hydroxyphthalide, (11) Oleoyl Ethanolamide and (12) Docosanedioic acid (Table 2). From remaining 36 compounds, limited information regarding retention time, molecular mass and formula of 23 compounds were determined, whereas for 13 compounds, only molecular mass and retention time were determined (Table 3). The 12 compounds identified from centipede haemolymph were subjected for novelty determination via Scifinder software. Interestingly, all of them were found to possess reported biological activities for their exact and homologous structures.

**Table 2:** Compounds identified from the red centipede haemolymph

No.	Compound	Formula	Structure	Reported activity
1	l-Homotyrosine	C10 H13 N O3		Exact structure: epithelial sodium channel blocker activity (Johnson 2015), antibacterial activity against *Pseudomonas aeruginosa* by inhibiting bacterial 4-hydroxyphenylpyruvate dioxygenase (Pascal et al. 1985), antifungal activity against *Candida albicans* and *Candida glabrata* by the inhibition of β-1,3-glucan synthesis (Klein et al. 2000; Zambias et al. 1992), act as matriptase inhibitors (Maiwald et al. 2016), antitumor activity (Ali et al. 2001), act as coagulation factor Xa inhibitors for treatment of cardiovascular diseases and thromboembolic events (Stürzebecher et al. 2015), antidiabetic activity (Bigge et al. 2003) Similar structure: antibacterial activity against *Staphylococcus aureus* (Or 1997), antifungal activity against *Candida* species (Hammond et al. 1992), antiprotozoal activity against *Trypanosoma b. rhodesiense* (Mehner et al. 2008), anticancer activity against HT-29 and HCT-116 colorectal cancer cells (Ooi et al. 2010; Mehner et al. 2008), used for the treatment of hyperlipidemia by cholesterol absorption inhibitory activity (Alenfalk et al. 2005), anti-diabetic activity (Bigge et al. 2003), used for the treatment of autoimmune disorders (Surolia et al. 2014)
2	8-Acetoxy-4-acoren-3-one	C17 H26 O3		Exact structure: this compound is the component of *Acorus calamus* (sweet flag) commonly found in spices (hmdb.ca), used for the treatment of epilepsy, amnesia and insomnia (Zhang et al. 2015), anti-germination activity (Nawamaki and Kuroyanagi 1996) Similar structure: growth inhibitory activity against *Staphylococcus aureus, Staphylococcus epidermidis, Bacillus cereus*, and *Escherichia coli* (Chuysinuan et al. 2019), antifungal activity against plant fungal pathogen *Pythium myriotylum* (Liu et al. 2016), *Phytophthora capsici* and *Pythium myriotylum* (Liu et al. 2015), anti-cancer activity against prostate carcinoma and human neuroblastoma cells (Wang et al. 2014), cytotoxic activity against human gastric cancer (BGC-823 cells), cervical cancer (Hela) and human alveolar basal epithelial cells (A549 cells) (Xu et al. 2014), pesticidal activity (Goldblum and Warren 2018)
3	*N*-Undecylbenzenesulfonic acid	C17 H28 O3 S		Exact structure: fungicidal activity against *Alternaria alternata*, *Chondrostereum purpureum*, *Phytophthora cactorum* and *P. infestans* (Komorowska-kulik et al. 1998), possess detergent property (Petrov et al. 1958; Matsunaga et al. 1996) Similar structure: antibacterial activity against *Staphylococcus aureus, Bacillus subtilis, Escherichia coli* and *Klebsiella pneumonia* and antifungal activity against *Aspergillus fumigatus* (Migahed et al. 2017), anti-tubercular activity against *Mycobacterium tuberculosis* H37Rv (Tanwar et al. 2016), pesticidal activity (Ichihashi and Okamura 2017; Hatamoto et al. 2016), fungicidal and herbicidal activity (Baba et al. 2014), act as UCH-L1 inhibitor useful for the treatment of cancer, Alzheimer disease and Parkinson disease (Lee et al. 2013), anticancer activity against human colon adenocarcinoma (Caco-2 cell line) (Rojewska et al. 2013), useful for the treatment of cancer and neurodegenerative disease (Lee et al. 2014)
4	2-Dodecylbenzenesulfonic acid	C18 H30 O3 S		Exact structure: act as agrochemical fungicides against *Venturia inaequalis*, *Botrytis cinerea*, *Erysiphe graminis, Phytophthora infestans*, and *Puccinia recondita* (Ihori et al. 2018), act as AKT PH domain inhibitors hence useful for the treatment of cancer (Ahad et al. 2011) Similar structure: antibacterial activity against *Staphylococcus aureus, Bacillus subtilis, Escherichia coli* and *Klebsiella pneumonia* and antifungal activity against *Aspergillus fumigatus* (Migahed et al. 2017), pesticidal activity (Ichihashi and Okamura 2017; Hatamoto et al. 2016), anti-tubercular activity against *Mycobacterium tuberculosis* H37Rv (Tanwar et al. 2016), act as sitagliptin (anti-diabetic agent) intermediates (Casar and Stavber 2014)
5	3*H*-1,2-Dithiole-3-thione	C3 H2 S3		Exact structure: commonly found in brassica (Human Metabolome Database), neuroprotective effects against PC12 (pheochromocytoma of the rat adrenal medulla) cells (Zhang et al. 2018a, b), used for the treatment of ischemic stroke and possess antioxidant and anti-inflammatory activity (Kuo et al. 2017), neurodegenerative activity (Brown et al. 2014), antiviral activity against human papilloma virus (Preston and Murphy 2015), antifungal activity against *Candida* species (Giannini et al. 2004), act as chemoprotective agent against cancer (Kwak et al. 2001), used for the treatment of autoimmune encephalomyelitis (Kuo et al. 2016) Similar structure: protective effects against Alzheimer’s disease (Wang et al. 2017a, b), antioxidant activity (Koo et al. 2012), used to prevent and treat a disease caused by over activity of a liver X receptor α (LXR α) (Kim et al. 2016), used for the treatment of skin pigmentation disorders (Commo and Michard 2009), neuroprotective activity (Jia et al. 2009), act as cancer preventive agent (Tran et al. 2009), antioxidant activity (Perez-Leal et al. 2017), anti-inflammatory and anti-neurodegenerative activity (Jarrott and Williams 2016)
6	Acetylenedicarboxylate	C4 H2 O4		Exact structure: act as succinate receptor agonists (Geubelle et al. 2017), act as inhibitors of bacterial urease released by *Helicobacter pylori* and *Proteus mirabilis* (Macegoniuk et al. 2017), used in the synthesis of quinoline and pyrroloquinoline derivative with anticancer activity against MCF-7 (breast cancer), HepG2 (liver carcinoma) and HCT (human colon cancer) cells (Mohamede et al. 2015), used in the synthesis of anticancer compounds against human gastric carcinoma N87 cells (Zhao et al. 2016), involved in the synthesis of anti-giardia and anti-HIV agent (Al-Masoudi and Abbas 2016), involved in the synthesis of alpha-glucosidase inhibitors (Hyun et al. 2014) Similar structure: antibacterial activity against Gram-negative bacteria such as *Pseudomonas aeruginosa* and *Escherichia coli* (Balkovec et al. 2017), involved in the synthesis of p53 inhibitors as anti-cancer and anti-inflammatory agent (Feder et al. 2015), involved in the preparation of amanita toxins which are effective in abnormal cell growth, proliferative disorder, neuronal disorders, immunological disorders, inflammatory disorders, autoimmune disorders, destructive disorders, bone disorder, infectious disease, neurodegenerative disorder, pancreatitis or kidney disease in a mammal (Zhao et al. 2017)
7	Albuterol	C13 H21 N O3		Exact structure: therapeutic agent for lymphedema (Hirata et al. 2018), used in the synthesis of anticancer agent against gastric carcinoma (Zhao et al. 2018), antidepressant activity (Avram et al. 2018), anti-inflammatory and anti-asthmatic effects (Lee et al. 2016; Hakonarson et al. 2018), used to treat cardiovascular diseases (Wang et al. 2018a, b, c), anti-diabetic activity (Pelcman and Bengtsson 2018) Similar structure: anti-epileptic activity (Stewart et al. 2018), anti-inflammatory and anti-asthmatic effects (Alvarez-Aguilar et al. 2017), used to treat Parkinson’s disease (Scherzer 2018), used for the treatment of hypoxemia and dyspnea (Martin 2018), anti-cancer activity (Weinstein et al. 2018), used to treat cardiovascular diseases (Wang et al. 2018a, b, c)
8	Tetradecylamine	C14 H31 N		Exact structure: bactericidal activity against *Staphylococcus aureus* and *Escherichia coli* (Niu et al. 2018; Savage Paul 2017), pesticidal activity (Park et al. 2018), anti-inflammatory activity (Wrasidlo and Natala 2018), antifungal activity against *Candida* and *Aspergillus* species by inhibiting ergosterol synthesis (Chandrika, et al. 2018; Garneau-Tsodikova et al. 2018), used as a component in traditional Chinese medicine for the treatment of Coronary heart disease complicated with depression (Zhang et al. 2018a, b) Similar structure: antibacterial activity against *Escherichia coli* (Wang et al. 2018a, b, c), anticancer activity against bladder cancer T-24 cells (Wu et al. 2017), involved in the synthesis of antimycobacterial agent (Vosátka et al. 2018); anti-tubercular activity (de Castro et al. 2018), anti-inflammatory activity (Wrasidlo and Natala 2018)
9	Curcumenol	C15 H22 O2		Exact structure: anti-inflammatory activity (Lee et al. 2019), antistroke agent with anti-inflammatory and cytotoxic activity for sepsis and leukemia, this compound is present in *Curcuma longa* (Turmeric) (Gupta et al. 2018), anti-proliferative activity against human gastric cancer cells (Jung et al. 2018), antibacterial activity against *Proteus mirabilis*, *Staphylococcus aureus* and antifungal activity against *Fusarium oxysporum* (Kacem et al. 2016) Similar structure: anti-skin inflammation activity (Lim et al. 2018), neuroprotective activity (Xu et al. 2018), anticancer activity against nasopharyngeal carcinoma cells (Wang et al. 2018a, b), larvicidal activity against *Aedes aegypti* larvae (Sofian et al. 2017), cytotoxic activity against human prostate carcinoma cells, human skin fibroblasts (HSF) and human melanoma cells (Stojakowska et al. 2019), antileukemic activities against the KG1a and Molt4 cell lines (Anuchapreeda et al. 2018), anti-fungal activity against *C. albicans* (Li et al. 2017), antioxidant, anti-inflammatory, anti-cancer, and anti-diabetic activity (Hamidpour et al. 2015), antimicrobial activity against *Klebsiella pneumonia*, *Staphylococcus aureus*, *Salmonella enterica*, *Escherichia coli*, *Pseudomonas aeruginosa*, *Proteus vulgaris*, and fungus *Pichia guilliermondii* and *Candida albicans* (Kharkwala et al. 2017)
10	3-Butylidene-7-hydroxyphthalide	C12 H12 O3		Exact structure: found in the roots of *Angelica sinensis* (AS) (Deng et al. 2006), anti-inflammatory activity (Tran et al. 2018), act as synergistic calcium antagonists for the treatment of coronary heart disease (Lei et al. 2018), cytotoxic activity against MCF-7 (breast cancer), NCI-H187 (lung cancer) and KB cells (Wisetsai et al. 2018), act as pancreatic lipase inhibitor for treatment of obesity (Mo et al. 2016), used for the treatment of peptic ulcer (Chung et al. 2005), used for the treatment and prevention of diabetes mellitus (D'orazio et al. 2007) Similar structure: free radical scavenging activity (Adil et al. 2018), active component of *Angelica sinensis* (AS) herb, used as the blood-nourishing tonic (Chen et al. 2017), anti-inflammatory activity (Tran et al. 2018), antioxidant and antibacterial activity against *Bacillus subtilis*, *Staphylococcus aureus*, *Escherichia coli*, *Pseudomonas aeruginosa*, *Klebseilla pneumonia*, *Agrobacterium tumefaciens* and antifungal activity against *Candida albicans*, *Mucor* sp., *Aspergillus flavus*, *Penicilium expansum* (Ksouri et al. 2017), neuroprotective effect on PC12 cells (Lu-Si et al. 2017), used to treat bone diseases (Wang et al. 2017a, b), prevents cancer by increase the oxygen release efficiency of Hb (Wang and Chen 2017), neuroprotective and anticancer effects against lung (A549), human colon carcinoma (HCT-8), and hepatocarcinoma (HepG2) cancer cell (Gong et al. 2016)
11	Oleoyl ethanolamide	C20 H39 N O2		Exact structure: endogenous peroxisome proliferator-activated receptor alpha (PPAR-α) agonist (Gaetani et al. 2003), antitussive activity (Wortley et al. 2017), anti-inflammatory activity (Toguri et al. 2018), used to treat post-traumatic stress disorder by fatty acid amide hydrolase (FAAH) inhibition (Danandeh et al. 2018), useful in the treatment of neurological disorders (Pandey et al. 2018), anti-nausea effect (Rock et al. 2017), analgesic activity (Zubrzycki et al. 2017), anticancer activity against colon cancer cells (Pagano et al. 2017) Similar structure: anti-inflammatory and pain-relieving effects (Britti et al. 2017), useful in the treatment of inflammatory and neurodegenerative disorders (Barbierato et al. 2018), anticancer activity against colon cancer cell growth (de Cedrón et al. 2018), beneficial in the treatment of HIV-1 associated neurocognitive disorders (HAND) (Hermes et al. 2018), anticancer activity against endometrial cancer (Fonseca et al. 2018), useful in the treatment of intestinal barrier dysfunction (Antón et al. 2018)
12	Docosanedioic acid	C22 H42 O4		Exact structure: plant metabolite with antifungal activity against *Candida albicans*, *Cryptococcus neoformans*, *Aspergillus fumigatus* and *dermatophyte Trichophyton rubrum* (Bierer et al. 1995; Bierer et al. 1998), anti-HIV activity (Brinkworth and Bairlie 1992), act as bivalent histamine H2 receptor (H2R) agonists (Birnkammer et al. 2012), synthesis study (Frost et al. 2010), anti-cancer and anti-inflammatory activity (Gao et al. 2013) Similar structure: antioxidant activity (Kaneria et al. 2018), skin pigmenting activity (Giuliani et al. 2015), antimalarial activity (Baba et al. 2015), deodorant component (Sato 2016), involved in the treatment of disorders including obesity and diabetes (Just et al. 2016), cosmetic component (Nomura et al. 2016)

**Table 3:** Compounds identified in the haemolymph of red-headed centipede

Compound label	Retention time	Molecular mass	Molecular formula
Cpd 1	0.546	244.90629	C3 H Cl2 N3 O4 S
Cpd 2	0.595	147.97314	ND
Cpd 3	14.311	267.11138	C13 H17 N O5
Cpd 4	18.808	340.20795	C19 H32 O3 S
Cpd 5	19.979	117.93689	ND
Cpd 6	20.119	845.95569	ND
Cpd 7	20.256	232.95286	ND
Cpd 8	20.309	135.90438	ND
Cpd 9	20.316	101.94352	ND
Cpd 10	20.329	145.93312	C4 H2 S3
Cpd 11	20.393	983.99919	ND
Cpd 12	20.484	230.91116	ND
Cpd 13	20.485	176.99131	C4 H3 N O7
Cpd 14	20.486	198.9733	C10 H N O2 S
Cpd 15	20.502	62.99858	ND
Cpd 16	20.533	201.86891	ND
Cpd 17	20.582	227.98881	C8 H4 O8
Cpd 18	20.942	1034.9965	ND
Cpd 19	0.554	161.0228	C7 H3 N3 O2
Cpd 20	0.586	63.00717	ND
Cpd 21	0.627	161.10154	C3 H11 N7 O
Cpd 22	0.84	203.1128	C5 H13 N7 O2
Cpd 23	12.338	227.18775	C13 H25 N O2
Cpd 24	14.833	295.21517	C17 H29 N O3
Cpd 25	15.584	346.24091	C16 H34 N4 O2 S
Cpd 26	16.695	524.3939	C28 H52 N4 O5
Cpd 27	16.72	56.06329	C4 H8
Cpd 28	16.752	148.01597	C8 H4 O3
Cpd 29	16.759	480.36669	C26 H48 N4 O4
Cpd 30	16.821	436.34066	C23 H48 O7
Cpd 31	16.875	392.31449	C22 H40 N4 O2
Cpd 32	18.324	386.27256	C27 H34 N2
Cpd 33	20.509	610.16105	C37 H27 Cl N4 O S
Cpd 34	21.305	701.20692	C44 H32 Cl N3 O4
Cpd 35	22.174	662.44722	C33 H58 N8 O6
Cpd 36	22.316	775.22523	ND

*ND* not determined

## Discussion

Development of robust antimicrobials from novel sources is the current need to counter drug resistant pathogens (Challinor and Bode 2015; Harvey et al. 2015). Most common sources of antimicrobials are bacteria, fungi, plant and plant products that have been used widely in modern medicine (Abraham et al. 1953; Wagman 1980; Negi et al. 1999). In contrast, discovery of antimicrobials from animal sources is an area explored superficially. This is despite the fact that animals particularly invertebrates such as cockroaches, ants, silk worms, scorpions and tarantulas have been used in traditional medicine for centuries (Costa-Neto 2005). For example, larval therapy is used widely to cure non-healing wounds. This involves, the application of mature blow fly larvae belonging to *Sarconesiopsis* genus on an open wound, resulting in the secretion of antimicrobial peptides and metabolites (Diaz-Roa et al. 2018). Maggot debridement therapy is effective to cure severe necrotizing fasciitis, caused by more than one type of bacteria such as MRSA, *Streptococcus pyogenes*, enterococci, *E. coli*, *P. aeruginosa*, *Clostridium* and *Bacteroides* species (Maya et al. 2014). Maggot debridement therapy is useful in patients suffering from necrotizing fasciitis with an underlying disease who cannot be subjected to surgical procedures such as diabetic patients (Dunn et al. 2002). Other studies showed that application of sterile larvae belonging to genus *Lucilia sericata*, *Protophormia terraenovae*, *Sarconesiopsis magellanica* secretes antimicrobial molecules/peptides such as as *p*-hydroxybenzoic acid, *p*-hydroxyphenylacetic acid, dioxopiperazine proline, seraticin, defensins, cecropins, diptericins and proline-rich peptides with potent anti-biofilm and wound healing properties (Nigam et al. 2010; Chernysh et al. 2018). Similarly, arthropods such as wild centipedes have been used in traditional Chinese medicine, often used to treat various illnesses such as seizures, apoplexy, stroke induced hemiplegia, diphtheria, tuberculosis, pyocutaneous disease etc. (Moon et al. 1996; Undheim and King 2011). In Korea, crushed centipede is used to treat back pains, sores and furuncles (Douglas 2014). Recent studies also highlight its broad range of antimicrobial activity against various pathogens. For example, *S. subspinipes mutilans* exhibited antifungal activity by membrane permeabilization in *Candida albicans* (Choi et al. 2013). Similarly, antimicrobial activity of the peptide lacrain, isolated from body extract of *S. viridicornis* showed strong bactericidal activity against Gram-negative bacteria (Chaparro and Da Silva Junior 2016). 3,8-Dihydroxyquinoline also known as jineol, isolated from *S. subspinipes mutilans* showed antibacterial activity by altering the release of potassium ions from food borne pathogenic strains of *E. coli* O157:H7 and *S. aureus* KCTC-1621 (Bajpai et al. 2017). Several other AMPs such as Scolopendrasin I, V, VII are known to possess broad range of antimicrobial activities against drug resistant pathogens (Wenhua et al. 2006; Peng et al. 2010). For the first time, here we determined the antibacterial activity of the haemolymph/organ lysates of red-headed centipede *S. subspinipes*, with molecular identification of biological components using LC/MS. Our findings suggest that haemolymph and tissue extract of centipede exhibited antibacterial activity against Gram-positive and Gram-negative bacteria. Haemolymph subjected to chemical characterization indicated the identification of 12 compounds with reported biological activities against Gram-positive and Gram-negative bacteria, fungi, viruses and parasites (Pascal et al. 1985; Komorowska-kulik et al. 1998; Niu et al. 2018; Bierer et al. 1998; Baba et al. 2015). For example, compounds 1, 3, 4, 5, 6, 8, 9 and 12 possess antimicrobial activity against a broad range of microorganisms such as *S. aureus*, *P. aeruginosa*, *P. mirabilis E. coli*, *H. pylori*, *Aspergillus* species*, Candida* species, *F. oxysporum, C. neoformans, dermatophyte T. rubrum, A. alternata*, *C. purpureum*, *P. cactorum*, *P. infestans*, *V. inaequalis*, *B. cinerea*, *E. graminis, P. recondite,* Human Papilloma virus, HIV and parasite Giardia.

Moreover, compound**s** 1, 4, 5, 6, 7, 9, 10, 11, 12 possess anticancer activity against colon cancer cells, MCF-(breast cancer), NCI-H187 (lung cancer) and KB cells, human gastric cancer cells, HepG2 (Liver carcinoma) cells (Pagano et al. 2017; Wisetsai et al. 2018; Jung et al. 2018; Ali et al. 2001; Bigge et al. 2003; Kuo et al. 2016; Pelcman and Bengtsson 2018; Lee et al. 2016; Hakonarson et al. 2018) (Table 2).

Interestingly, some of the compounds identified also possess antidiabetic, anti-neurodegenerative, antioxidant, antiepileptic and anticancer activities (Bigge et al. 2003; Wisetsai et al. 2018; Gong et al. 2016). Identified compounds contain furan, tyrosine, thione, albuterol, amines, curcumenol and pthalide moieties, potentially responsible for biological activities. Notably, compounds 2, 5, 9, 10 and 12 are phytochemicals with antibacterial, antifungal, anti-inflammatory, anticancer and analgesic properties (Giannini et al. 2004; Gupta et al. 2018; Tran et al. 2018; Kacem et al. 2016; Brinkworth and Bairlie 1992). Biological significance of these compounds are due to their distinct features and structural arrangement of the functional groups. For example, sulfides and disulfides in cpd 5 are active ingredients. Sulphur has its characteristic property and is an essential component in antibiotics such as sulphonamides (Mitchard 1988). Curcumenol cpd 9, containing tetrahydrofuran is an active five-membered oxygen heterocyclic compound, commonly found in natural products, mainly responsible for their antibacterial activity (Keglevich 2015). Phthalides and fatty acids present in cpd 10 and 12 are also well known for their broad spectrum activities such as antiinflammatory, antimicrobial and anticancer activities (Bierer et al. 1995; Gao et al. 2013; Wisetsai et al. 2018). Notably, 36 compounds were not identified in this study. These are also of potential interest and could represent novel antibacterials (Table 3).

In summary, the discovery of natural antibiotic molecules from animals/invertebrates, exposed to the environmental wastes and pollutants in their natural habitat is a fascinating though unexploited area of research. Hence, it is anticipated that the antibiotics from natural sources are minimal or less toxic for biological system as compared to synthetic antibiotic molecules. Further identification, in vivo testing and clinical trials of potentially active metabolites can act as a milestone for the synthesis and development of novel drug leads.

## Data Availability

All the data analysed in this study are included in this article.

## Abbreviations

MRSA: methicillin resistant *Staphylococcus aureus*
MTCC: Malaysian Type Culture Collection
ATCC: American Type Culture Collection
EDTA: ethylenediamine tetraacetic acid
cfu: colony forming units
Hacat: human keratinized skin cells
RPMI: Roswell Park Memorial Institute
LDH: lactate dehydrogenase
LC–MS: liquid chromatography–mass spectrometry
Q-TOF: quadrupole-time of flight
ESI: electrospray ionization
